# Resistance Training Before Hyperalgesia Induction Promotes Analgesic Effects Through Central and Peripheral Biomarker Modulation in an Experimental Fibromyalgia-like Model

**DOI:** 10.3390/life15060849

**Published:** 2025-05-24

**Authors:** Andrês Valente Chiapeta, Leandro Licursi de Oliveira, Luciano Bernardes Leite, Bruna Aparecida Fonseca da Silva, Sebastião Felipe Ferreira Costa, Leôncio Lopes Soares, Alexa Alves de Moraes, Lucas Rios Drummond, Pedro Forte, Antônio José Natali, Miguel Araujo Carneiro-Júnior

**Affiliations:** 1 Laboratory of Exercise Biology, Department of Physical Education, Federal University of Viçosa, Viçosa 36570-900, Brazil; andreschiapeta@gmail.com (A.V.C.); luciano.leite@ufv.br (L.B.L.); bruna.a.silva@ufv.br (B.A.F.d.S.); sebastiao.costa@ufv.br (S.F.F.C.); leoncio.lopes@ufv.br (L.L.S.); alexa@ufv.br (A.A.d.M.); anatali@ufv.br (A.J.N.); miguel.junior@ufv.br (M.A.C.-J.); 2 Laboratory of Structural Biology, Department of General Biology, Federal University of Viçosa, Viçosa 36570-900, Brazil; leandro.licursi@ufv.br; 3 Department of Physiology and Biophysics, Federal University of Minas Gerais, Belo Horizonte 30130-100, Brazil; lucas.rios.drummond@gmail.com; 4 Physical Education Department, State University of Minas Gerais, Divinópolis 35500-000, Brazil; 5 Research Center for Physical Activity and Wellbeing (Livewell), Instituto Politécnico de Bragança, 5301-857 Bragança, Portugal; 6 Department of Sports, Higher Instituto of Educational Sciences of the Douro, 4560-547 Penafiel, Portugal; 7 Department of Sports Sciences, Instituto Politécnico de Bragança, 5301-857 Bragança, Portugal

**Keywords:** physical exercise, chronic pain, cytokines, serotonin

## Abstract

Background: Fibromyalgia is a chronic syndrome characterized by widespread pain and complex pathophysiology, requiring new therapeutic approaches. This study aims to investigate the effects of resistance training (RT) before hyperalgesia induction on pain sensitivity, IL-6 and IL-10 expression in skeletal muscle, and thalamic serotonin levels in a fibromyalgia (FM)-like rat model. Methods: Wistar female rats aged 12 months were divided into four groups: untrained neutral saline (UNS), untrained acid saline (UAS), RT neutral saline (RTN), and RT acid saline (RTA). Both the RTN and RTA groups were subjected to an RT protocol comprising vertical ladder climbing three times a week throughout 14 weeks. The UAS and RTA groups received 100 µL of neutral, sterile saline (pH 4.0) in the left gastrocnemius muscle, while the UNS and RTN groups received 100 µL of neutral saline (pH 7.4). Mechanical hyperalgesia was assessed using Von Frey’s electronic esthesiometer. Expression of interleukins 6 (IL-6) and 10 (IL-10) was analyzed in skeletal muscle, and serotonin expression was quantified in the thalamus. Results: After hyperalgesia induction, the RT groups demonstrated higher paw withdrawal thresholds than the UAS group (*p* < 0.05). Both IL-6 and IL-10 expression was lower in the RTA group compared to the UAS group (*p* < 0.05). Thalamic serotonin expression was higher (*p* < 0.05) in the RTA group compared to the UAS group. Conclusion: Previous RT was able to prevent mechanical hyperalgesia experienced by rats after its induction by acid saline by preventing the increase in the IL-6 and IL-10 levels in skeletal muscle and preventing the decrease in thalamic serotonin expression.

## 1. Introduction

Fibromyalgia (FM) is characterized by persistent and diffuse muscle pain and fatigue, which is usually accompanied by physical, cognitive, and psychological disturbances, and it does not have a healing treatment so far [1,2,3]. It affects about 0.2 to 6.6% of the world’s population, with women around 35 to 44 years old being more commonly affected [4,5]. Patients with FM usually present hyperactivation of pain-ascending signaling pathways and downregulation of pain-inhibitory descending pathways, and the causes of such dysregulation have not been well elucidated yet [6].

Some studies support the hypothesis that people with FM may present dysfunctional regulation of cytokine production. For instance, imbalances between pro- and anti-inflammatory cytokines (i.e., IL-6 and IL-10) may decrease the pain threshold and cause peripheral nerve hypersensitivity to nociceptive stimuli [7]. Although these patients may exhibit a particular cytokine profile implicated in pain modulation, the relationship between such cytokine expression patterns and clinical evidence of pain remains inconclusive [8].

Moreover, evidence suggests that FM also involves low serotonin levels, which is important in endogenous pain descending inhibitory pathways [9]. Thus, serotonergic dysfunction is a major hypothesis among FM causes, which is supported by the efficacy of drugs that regulate serotonin metabolism [2].

Regarding physical exercise training, it modulates the immunological system, which interferes with pain management. Therefore, inactive individuals present higher expression of inflammatory cytokines than anti-inflammatory ones, while active individuals display the opposite pattern. Additionally, it prevents hyperalgesia episodes through increased serotoninergic activity [10]. Nevertheless, few studies have explored the potential effect of physical exercise on preventing the development of chronic muscle pain, especially in FM-like models [11,12]. Although resistance training (RT) has emerged as a consistent clinical option for improving quality of life and functionality without exacerbating painful symptoms [13,14], none of these studies have tested RT as a potential hyperalgesia-prevention intervention, being restricted to aerobic protocols only.

Research using FM-like animal models has become increasingly relevant for uncovering mechanisms of exercise-induced analgesia, offering valuable guidance for clinical management [15,16]. These models allow the evaluation of specific exercise protocols and their effects on fibromyalgia-like symptoms, showing that aerobic and resistance training can alleviate pain and enhance quality of life [17,18]. Exercise training notably improves serotonin neurotransmission, a key factor in pain modulation [17]. Given the lack of consensus on preventing pain exacerbation in FM and the limited evidence on the preventive role of RT, this study investigates the effects of RT before hyperalgesia induction on pain sensitivity, IL-6 and IL-10 expression in skeletal muscle, and serotonin levels in the thalamus in a FM-like rat model. The cytokines IL-6 and IL-10 were selected because they represent key pro- and anti-inflammatory mediators involved in the nociceptive sensitization and immune dysregulation observed in fibromyalgia. The thalamus was chosen due to its central role in pain integration and descending inhibitory modulation via serotonergic neurotransmission, which is frequently altered in FM patients and targeted by exercise interventions. Therefore, the aim of this study was to evaluate the effects of an RT protocol prior to hyperalgesia induction on pain sensitivity, IL-6 and IL-10 expression in skeletal muscle, and thalamic serotonin levels in an FM-like rat model. It was hypothesized that the RT increased physical performance and prevented the increase in pain sensitivity.

## 2. Materials and Methods

### 2.1. Animals and Study Design

**Figure 1:**
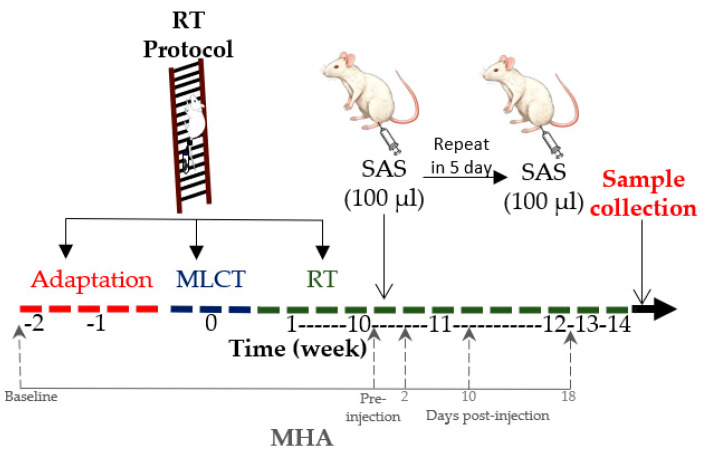
Flowchart of the study design. RT: resistance training; MLCT: maximum load carrying test; SAS: subcutaneous acid saline; MHA: muscle hyperalgesia model.

This experimental, preclinical study used an animal model to simulate FM-related pain and investigate the effects of resistance training on pain sensitivity and inflammatory response. Rats were assigned to different experimental groups and subjected to controlled interventions, including a resistance training protocol and hyperalgesia induction. Considering that FM mostly affects middle-aged women [2,5], 12-month-aged female Wistar rats were assigned to the following groups, with *n* = 8 in each of them: UNS (untrained neutral saline), UAS (untrained acid saline), RTN (resistance training neutral saline) and RTA (resistance training acid saline). The animals were housed in cages containing 4 rats each and kept in a room with controlled temperature (22 ± 2 °C) under 12/12 h light/dark cycles and free access to water and standard rodent chow. All procedures were carried out following the National Guidance for the Care and Use of Laboratory Animals and had the consent of the Institutional Ethics Committee (CEUA-UFV 31/2020). Figure 1 presents a flowchart of the study design.

### 2.2. Resistance Training

The RTN and RTA groups were submitted to a vertical ladder climbing RT protocol divided into 3 phases [19]: adaptation, maximum load carrying test, and the 14-week RT protocol. After the 10th training week, the animals were subjected to hyperalgesia induction. 

#### 2.2.1. Adaptation Phase

A two-week adaptation to the RT protocol was carried out three times per week so that the animals could become familiar with the equipment. It comprised a vertical ladder (1.1 × 0.18 m, 2 cm between the steps, inclination at 80°), and each rat was initially positioned at the top of the ladder, inside a polycarbonate cage, for five minutes. After this period, the animals were positioned at the bottom of the ladder and stimulated to climb it three times, with a two-minute rest between each climb. In the first week, no extra load was added to each climb. In the second one, a device was attached to the proximal portion of the animals’ tails, which would receive weights for the RT protocol.

#### 2.2.2. Maximum Load Carrying Test

The maximum load carrying test comprised four to eight climbs with progressively heavier loads and a two-minute rest between each climb. In the first attempt, 75% of the animal’s body weight was applied. If the animal could carry it, an additional weight of 30 g was added to the device. This procedure was successively repeated until the animal failed to reach the top of the ladder. The value considered as the maximum carrying load was the one that allowed the animal to complete the climb without failing (i.e., the penultimate attempt).

#### 2.2.3. Resistance Training Protocol

Following the completion of the maximum load test, the Wistar rats were subjected to a 14-week RT protocol, which was conducted three times a week—specifically, on Mondays, Wednesdays, and Fridays—at the same time each day. In the initial week, the rats performed two climbing sessions, and from the second week onward, they engaged in three climbing sessions per training day. Each climbing session involved the application of the maximum load, with a two-minute rest period between each attempt to allow for recovery and optimal performance. After the fourth week, a new maximum load carrying test was administered to readjust the load for each animal, thus ensuring that the training intensity remained appropriate throughout the study.

### 2.3. Hyperalgesia Induction

Hyperalgesia was induced as proposed by Sluka et al. [10], which mimics FM pain. After the end of the 10th week of the RT protocol, the animals rested for 24 h before the hyperalgesia induction.

All experimental groups underwent anesthesia using isoflurane inhalation at a 3% concentration with 100% FiO2, allowing for instinctive ventilation during the procedure [20]. The UAS and RTA groups received 100 µL of sterile acidic saline (pH 4.0) injection into the left gastrocnemius muscle. By contrast, the UNS and RTN groups were injected with the same volume of neutral saline solution (pH 7.4). This injection protocol was repeated after five days, resulting in a second round of animal injections.

Administering acidic saline is known to induce bilateral mechanical hyperalgesia, closely mimicking the pain experienced in fibromyalgia (FM) and persisting for approximately 30 days [21]. After inducing hyperalgesia, the resistance training protocol for the trained groups continued for an additional four weeks, which allowed for the assessment of the effects of exercise on pain sensitivity and recovery [22]. This model effectively simulates the chronic pain conditions associated with fibromyalgia, providing a valuable framework for investigating potential therapeutic interventions [23].

### 2.4. Mechanical Hyperalgesia Assessment

Mechanical hyperalgesia was evaluated at five distinct time points: before the initiation of the exercise protocol (baseline), immediately before the induction of hyperalgesia, and subsequently at 2, 10, and 18 days following the injections. This assessment was conducted using an electronic esthesiometer (Von Frey digital—Insight Research and Teaching, Ribeirão Preto—SP, Brazil), which quantifies pain thresholds based on the withdrawal response of the hind paw to a mechanical pressure stimulus. All evaluation sessions were performed in a controlled environment, characterized by silence and a stable temperature, ensuring consistency across assessments. A single-blinded researcher conducted all measurements at the same time of day to eliminate variability [24].

Following a 30-min acclimatization period to the testing apparatus, each animal was subjected to five pressure stimuli applied to each paw, with a 30-s interval between stimuli to allow recovery [25]. The paw withdrawal threshold was recorded based on the animals’ reactions, which included paw withdrawal, licking, or jumping in response to the applied pressure. After completing the five assessments for each paw, the median threshold for each animal’s paw was calculated. The mean deviation was then determined for each result, and the three values with the lowest mean deviation were utilized to establish the mean paw withdrawal threshold. In this study, the paw withdrawal thresholds were presented as an average that encompassed both paw sides, thus providing a comprehensive measure of mechanical hyperalgesia [26].

### 2.5. Sample Collection

Two days after the final training session, the animals were euthanized through decapitation [27]. Brain and gastrocnemii muscle were quickly withdrawn and stored at −80 °C. This immediate freezing is crucial, as it prevents the degradation of proteins and nucleic acids, which can occur rapidly post mortem, thereby ensuring the reliability of any molecular assessments conducted later [28,29]. The gastrocnemius muscle, in particular, is of interest due to its role in locomotion and the fact that it was the site of acid saline injection, which makes it a relevant target for evaluating local inflammatory responses and the effects of exercise on muscle pain and recovery [30]. Brain samples are equally important, as they allow for the exploration of the central mechanisms underlying pain modulation and the potential neurobiological adaptations resulting from exercise intervention [31].

### 2.6. Muscle IL-6 and IL-10 Determination

Gastrocnemius muscle samples were carefully excised and immediately weighed on an analytical balance. For each sample, 100 mg of tissue was placed in a pre-chilled microcentrifuge tube and homogenized on ice in 1 mL of phosphate-buffered saline (PBS, pH 7.4) supplemented with a protease inhibitor cocktail (Sigma-Aldrich, St Louis, MO, USA), using an OMNI motorized homogenizer (OMNI International, Kennesaw, GA, USA). Following homogenization, the samples were centrifuged at 10,000× *g* for 10 min at 4 °C. The resulting supernatant was carefully collected and aliquoted and then stored at −80 °C until further analysis to prevent protein degradation. Quantification of IL-6 and IL-10 concentrations in the muscle extracts was carried out using commercially available ELISA kits validated for rat IL-6 (ELK Biotechnology, Cat. No. ELK1158) and rat IL-10 (ELK Biotechnology, Cat. No. ELK1144), following the manufacturer’s instructions. Absorbance was measured at 450 nm using a microplate spectrophotometer (BioTek Synergy HTX Multi-Mode Reader, Miami, FL, USA). Cytokine concentrations were calculated based on standard curves generated for each assay and normalized to the initial tissue weight, with results expressed as ng/mg of tissue. All sample processing and analyses were conducted in a blinded manner concerning experimental group allocation to minimize bias.

### 2.7. Thalamic Serotonin Determination

The thalamus was chosen due to its important role in pain perception [32]. It was removed from the animal’s brain using a cryostat (Leica CM 1850) with a 60-micrometer-section thickness, and the brain was placed inside of the cryostat at a temperature of −20 °C. To precisely locate the thalamic region, a rat brain anatomy atlas was used [33]. The tissue was removed using a 2 mm Kai Punch needle, and after removal, the material was stored at −80 °C. Serotonin expression was analyzed using the competitive ELISA, using the commercial kit Elabscience^®^ ST/5-HT E-EL-0033 (Houston, TX, USA), following the manufacturer’s instructions. The concentrations were expressed in ng/thalamus.

### 2.8. Statistical Analysis

Data were checked for normality using the Kolmogorov–Smirnov test. The intergroup comparisons regarding maximum carrying load, mechanical hyperalgesia, muscle IL-6 and IL-10, and thalamic serotonin were carried out through a two-way ANOVA followed by Tukey’s post hoc. The effect size (ES) was reported to emphasize the magnitude of difference between the groups, being expressed as eta squared (η^2^): small (0.01 < η^2^ 0.06); medium (0.06 ≤ η^2^ < 0.14); and large (η^2^ ≥ 0.14) [34]. The ES considered the factors of acid saline, resistance training, and interaction between both factors. Data are presented as mean ± standard deviation (SD), and *p* < 0.05 was considered statically significant. All analyses were conducted in the statistical program SigmaPlot, version 14.5.

## 3. Results

### 3.1. Mechanical Hyperalgesia

Table 1 presents the mean paw withdrawal thresholds of the animals throughout the experiment days. Paw withdrawal threshold did not exhibit any difference between groups (*p* > 0.05) on days 0 (baseline; η^2^ = 0.09) or 70 (pre-induction; η^2^ = 0.00). However, on day 73, two days after pain induction, the acid saline significantly decreased (*p* < 0.05) the paw withdrawal thresholds in both the RTA and UAS groups, with a large effect size (η^2^ = 0.8) compared to the control groups. Also, the RTA group presented a higher paw withdrawal threshold (*p* < 0.05) compared to the UAS group. The same pattern was observed on day 81, ten days after pain induction. Thus, the RT protocol prevented the increase in the pain sensitivity in the trained groups, with a large ES (*p* < 0.05; η^2^ = 0.6), while the UAS group presented the lowest paw withdrawal threshold compared to the other experimental groups (*p* < 0.05). Finally, on day 89, eighteen days after pain induction, the UAS group presented the lowest paw withdrawal threshold compared to the RTA and UNS groups (*p* < 0.05; η^2^ = 0.03).

### 3.2. Physical Performance

Table 2 presents the values of the maximum load carrying test for all experimental groups before the beginning of the resistance training (baseline) and 10 weeks after it, right before pain induction. At baseline, no intergroup differences were observed (*p* > 0.05; η^2^ = 0.03). However, after 10 weeks of the RT program, the RTN and RTA groups presented higher physical performance (*p* < 0.05) than the untrained animals, with a large ES (η^2^ = 0.68). Intergroup analysis revealed that the RTA group presented higher maximum load carrying test results than the RTN group prior to pain induction.

### 3.3. IL-6 and IL-10 in the Gastrocnemius Muscle

**Figure 2:**
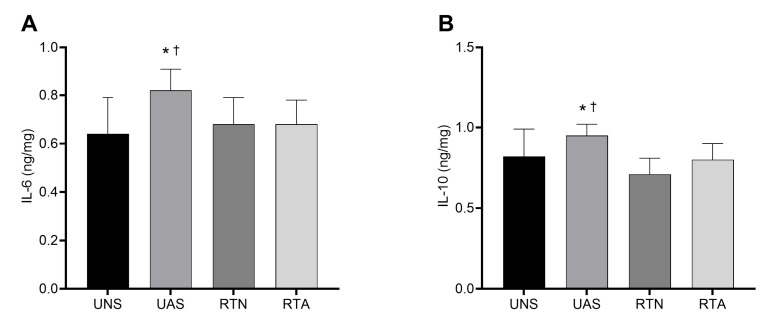
(**A**) Concentration of muscle IL-6. (**B**) Concentration of muscle IL-10. Values expressed as mean ± SD. UNS: untrained neutral saline group; UAS: untrained acid saline group; RTN: resistance training neutral saline group; RTA: resistance training acid saline group. * *p* ˂ 0.05 vs. UNS; ✝ *p* ˂ 0.05 vs. RTA.

Figure 2 presents the effects of RT and pain induction through acid saline in muscle concentrations of IL-6 (Figure 2A) and IL-10 (Figure 2B) at the end of the experiment. Acid saline injection increased IL-6 expression in the UAS and RTA groups compared to the neutral saline groups, with a large ES (*p* < 0.05; η^2^ = 0.14). The RTA group presented a lower concentration of IL-6 in relation to the UAS group (*p* < 0.05), which suggests that RT prevented the increase in the IL-6 concentration in the animals who were subjected to pain induction. Additionally, IL-6 expression in the RTA group did not differ from that in the RTN group. With regard to IL-10 expression, a similar pattern was observed. The UAS group exhibited higher IL-10 levels than the UNS group (*p* < 0.05), while the RTA group showed reduced IL-10 concentrations compared to the UAS group (*p* < 0.05), with a large effect size (η^2^ = 0.19).

### 3.4. Serotonin in the Thalamus

**Figure 3:**
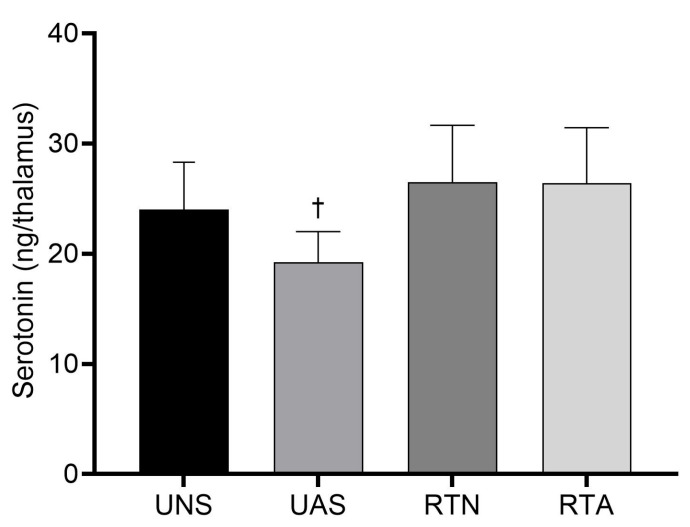
Concentration of serotonin in thalamus. Values expressed as mean ± SD. UNS: untrained neutral saline group; UAS: untrained acid saline group; RTN: resistance training neutral saline group; RTA: resistance training acid saline group. ✝ *p* ˂ 0.05 vs. RTA.

Figure 3 presents the concentration of serotonin in the thalamus at the end of the experiment. The UAS group presented a lower thalamic serotonin concentration compared to the RTA group (*p* < 0.05), with a large effect size (η^2^ = 0.26), which suggests that RT prevented the decrease in the trained animals. Also, serotonin levels in the UAS group displayed a downward tendency compared to the UNS group (*p* = 0.07).

## 4. Discussion

The present study explored the effects of an RT protocol before hyperalgesia induction on pain sensitivity, IL-6 and IL-10 expression in skeletal muscle, and thalamic serotonin levels in an FM-like rat model. There is still a lack of studies exploring the preventive effect of physical exercise on FM, especially RT protocols [11,12]. The results evidenced that RT increased physical performance, prevented the increase in pain sensitivity after hyperalgesia induction, prevented the thalamic serotonin reduction, and prevented the increase in both IL-6 and IL-10 levels in the gastrocnemius muscle.

The FM-like model induced by acid saline provoked mechanical hypersensitivity from the second to the eighteenth day after pain induction, with a large ES. This finding is supported by the significantly higher pro-nociceptive expression of muscle IL-6 in the untrained animals with acid saline, which sensitizes muscle nociceptors and results in hyperalgesia [7]. Moreover, IL-6 overexpression suggests a higher activation of the hypothalamic–pituitary–adrenal (HPA) axis, whose maladaptive hyperactivity is often seen in chronic pain disorders such as FM [35]. The UAS group also presented augmented levels of IL-10, which plays an anti-inflammatory role by inhibiting pro-inflammatory cytokines, such as IL-1, IL-6, and TNF-α. In the present study, such biomarker expression may have increased as a response to the IL-6 release. Nevertheless, the anti-inflammatory effect may not have prevented or even reversed the hyperalgesia outcome [36]. These results are in line with other experimental models of injury, such as spinal cord trauma, where IL-6 levels were also elevated and modulated in response to therapeutic interventions [37].

Additionally, thalamic serotonin expression was decreased in the untrained animals with acid saline. These findings corroborate previous studies, in which the acid saline-induced chronic pain model reduces the central concentration of serotonin, indicating a downregulation of pain descending pathways [11,38,39]. Thus, an imbalance between ascending and descending modulatory mechanisms may have resulted in augmented mechanical hypersensitivity.

Regarding exercise training, physical inactivity may be a triggering mechanism for chronic FM pain, and physical exercise seems to be a preventive and therapeutic option for such diseases, as it modulates both dopaminergic and serotonergic neurotransmission [11,40,41]. In this study, the 14-week RT protocol prior to hyperalgesia induction effectively prevented pain exacerbation and restored pain tolerance to the degree observed in the control animals. Two days after acid saline application, the RTA animals presented lower paw withdrawal thresholds than the controls. However, they were still higher than those of the UAS group. From day 10 until the end of the experiment, no differences between the RTA group and the controls were evident, while the UAS animals remained with lower pain tolerance levels. These results suggest a possible preventive effect of RT on pain sensitivity.

The exercise protocol was also effective in preventing the increase in muscle IL-6 and IL-10 levels in the RTA group compared to the untrained animals with acid saline, with values similar to those observed in the control groups. These findings reinforce the modulatory role of resistance training in inflammatory responses at the muscular level, particularly in conditions of nociceptive challenge. This effect is consistent with previous evidence demonstrating that regular physical exercise reduces serum [42,43,44] and muscle concentrations of pro-inflammatory mediators, including IL-6, in chronic pain conditions [45,46]. Such reductions are especially relevant in fibromyalgia, where a persistent elevation of these mediators contributes to increased pain perception. In this context, the inhibition of nociceptive cytokines by exercise has been proposed as one of the mechanisms underlying its protective effect, as it may attenuate the exaggerated activation of receptors associated with the ascending pain pathways [47]. Therefore, the maintenance of basal levels of IL-6 and IL-10 in the trained animals exposed to the pain model suggests that resistance training promoted an anti-inflammatory adaptation capable of limiting the development of hyperalgesia.

In addition, the low muscle concentrations of IL-10 in the trained groups may be explained by the anti-inflammatory effect of RT before hyperalgesia induction [47]. Thus, the low levels of IL-6 in such animals may not have been sufficient to activate the compensatory IL-10 release. This finding suggests that the RT protocol prevented muscle inflammatory response, reducing pain sensitivity in the animals who underwent physical exercise training before pain induction.

Another important finding was the preventive effect of exercise on pain modulation through the activation of serotonergic neurons within the descending inhibitory pathway. This was supported by the preservation of thalamic serotonin levels in the trained animals with acid saline, which were similar to those observed in the control animals. Previous studies have already demonstrated the effect of exercise training on upregulating serotonin levels [48,49], which culminates in the descending inhibition of nociceptive signaling. Thus, the RT protocol effectively prevented the changes caused by the pain induction, modulating peripheral inflammatory biomarkers on the pain ascending pathways and at a descending inhibitory level.

Finally, this study presents some limitations that should be acknowledged. First, we did not assess circulating cortisol levels, which could have provided important insights into the activation of the HPA axis in response to interleukin release. This would have helped confirm the involvement of neuroendocrine mechanisms often associated with stress and chronic pain modulation. Second, the measurement of substance P was not included, although it is a relevant neuropeptide involved in nociceptive transmission and neurogenic inflammation. Its quantification could have enhanced our understanding of the interaction between peripheral inflammation and central serotonergic modulation, particularly within the descending pain inhibitory pathway. Furthermore, our model focused exclusively on female rats, which is relevant given the higher prevalence of fibromyalgia in women; however, it limits the generalizability of the findings to males. Additional neurochemical and behavioral evaluations could enrich the characterization of the model and help clarify the proposed protective effect of resistance training. Therefore, we encourage future studies to incorporate these complementary assessments, including HPA axis markers and neuropeptides, as well as to explore different exercise protocols varying in volume, intensity, and frequency to refine our understanding of exercise-induced analgesia in fibromyalgia models.

## 5. Conclusions

In conclusion, a 14-week RT protocol before hyperalgesia induction by acid saline was able to prevent mechanical hyperalgesia in an FM-like female rat model by preventing the increase in the IL-6 and IL-10 levels in skeletal muscle and preventing the decrease in thalamic serotonin expression. This study indicates a protective effect of RT in attenuating the deleterious effects of chronic pain present in diseases such as fibromyalgia.

## Figures and Tables

**Table 1:** Paw withdrawal threshold (g) at baseline (day 0) and at subsequent time points after acid saline injection and/or resistance training.

Time	UNS	UAS	RTN	RTA	η^2^
0	37.25 ± 4.63	37.43 ± 4.54	39.47 ± 3.46	40.81 ± 4.69	0.09
70	40.75 ± 3.20	41.99 ± 1.63	41.75 ± 3.51	40.55 ± 2.57	0.00
73	44.66 ± 3.24	24.63 ± 4.33 *#✝	41.27 ± 2.34	32.58 ± 4.87 *#	0.80
81	43.03 ± 3.38	30.28 ± 3.46 *#✝	39.32 ± 2.10	39.28 ± 1.78	0.60
89	42.04 ± 2.58	37.81 ± 3.21 *✝	39.12 ± 1.97	41.15 ± 3.57	0.03

Values expressed as mean ± SD. UNS: untrained neutral saline group; UAS: untrained acid saline group; RTN: resistance training neutral saline group; RTA: resistance training acid saline group. * *p* ˂ 0.05 vs. UNS; # *p* ˂ 0.05 vs. RTN; ✝ *p* ˂ 0.05 vs. RTA.

**Table 2:** Maximum load carrying test values (g) at baseline (day 0) and after 10 weeks of resistance training (day 70).

Time	UNS	UAS	RTN	RTA	η^2^
0	308.1 ± 27.5	325.3 ± 61.7	327.4 ± 45.0	338.0 ± 54.0	0.03
70	336.3 ± 46.3	331.8 ± 22.5	421.3 ± 40.9 *&	462.0 ± 42.0 *&#	0.68

Values expressed as mean ± SD. UNS: untrained neutral saline group; UAS: untrained acid saline group; RTN: resistance training neutral saline group; RTA: resistance training acid saline group. * *p* ˂ 0.05 vs. UNS; & *p* ˂ 0.05 vs. UAS; # *p* ˂ 0.05 vs. RTN.

## Data Availability

The original contributions presented in this study are included in the article; further inquiries can be directed to the corresponding author.
